# Systematic Literature Review: Indoor Lighting and Color Effects on Persons With ASD

**DOI:** 10.1177/19375867251373096

**Published:** 2025-10-23

**Authors:** Veronika Zaikina, Hanne-Mari Schiøtz Thorud, Susanne Færdi Rustad, Helle Kristine Falkenberg

**Affiliations:** 1 Faculty of Health and Social Sciences, Department of Optometry, 11310 Radiography and Lighting Design, University of South-Eastern Norway, Kongsberg, Norway

**Keywords:** lighting design, autism, color, inclusive environment, interior design

## Abstract

**Aim:**

This systematic literature review, following the PRISMA statement, aims to review the knowledge of how the indoor lighting environment and color palettes impact individuals living with Autism Spectrum Disorders (ASD), particularly their behavior and lighting and/or color preferences.

**Background:**

A supportive built environment is crucial for persons with ASD. Lighting design (daylight and electrical lighting) and color schemes significantly impact their behavior, information processing, and overall well-being. Despite its importance, lighting design for autism has received limited attention in architecture and design research.

**Methods:**

A comprehensive search across seven electronic databases (PubMed, CINAHL, SveMed+, and four library databases including Oria, Regina, the British National Bibliography, and the Royal Danish Library), followed by a thorough review and critical appraisal, resulted in seven (7) high-quality studies with moderate to low risk of bias. Articles were assessed using three standardized checklists, for example, JBI Critical Appraisal Checklist for Analytical Cross-Sectional Studies, JBI Critical Appraisal Checklist for Qualitative Research, and Mixed Methods Appraisal Tool (MMAT).

**Conclusions:**

The findings are consistent with previous research and confirm that light and color influence ASD individuals’ behavior and sensitivity. However, there is a substantial gap in understanding practical applications, as most studies are descriptive or exploratory rather than experimental. Future research should emphasize experimental approaches to develop evidence-based guidelines for designers.

## Introduction

Designers have a critical responsibility in creating inclusive environments, an increasingly recognized concept within architecture and interior design, particularly for persons with autism spectrum disorder (ASD) (Clouse et al., 2020; Steele & Ahrentzen, 2015). Persons with ASD along with their families, educators, and caregivers, face unique challenges in navigating everyday environments, such as their homes, schools or workplaces. The Act on Equality and Prohibition of Discrimination (Lov om likestilling og forbud mot diskriminering, LOV-2017-06-16-51) anchored in European and international legislation (European Commission, 2019; United Nations, 2008), requires universal design for all public and private facilities. However, these legislative requirements often fail to sufficiently specify the needs of individuals with cognitive and sensory impairments, including those with ASD (Smedslund et al., 2023). National guidelines on lighting and color design for neurodiverse population varies considerably across countries, and there is no single, unified approach. Some countries, however, appear to be at the forefront of this development. For instance, the British Standards includes specific guidance on lighting and color for neurodiversity in the built environment (British Standards Institution, 2022). Similarly, International WELL Building Standard incorporates criteria addressing lighting, mental health, and cognitive well-being (International WELL Building Institute, 2024). The recommendations they provide are broad and open to interpretation. As such, they may not fully address the practical challenges designers face when attempting to apply them in real-world contexts (Dobstaff, 2024; Guo et al., 2024; Metz, 2021). This shortcoming, compounded by limited awareness and knowledge among designers, frequently results in environments that are not fully accessible, safe, or comfortable, causing unnecessary exclusion for this population. Thus, there remains a pressing need for more comprehensive, evidence-based guidelines on inclusive design addressing a broader spectrum of user needs and disabilities.

ASD describes a range of neurodevelopmental conditions with complex causes (American Psychiatric Association, 2013; Weidle, 2024). The current ICD-11 diagnostic system uses ASD as an umbrella term (World Health Organization, 2022). The ASD term “spectrum” refers to the wide variation in the presentation and severity of core characteristics—particularly difficulties with social interaction and communication, and restricted or repetitive patterns of behavior, interests, or activities (American Psychiatric Association, 2013; Weidle, 2024; World Health Organization, 2022). While it is not part of the diagnostic criteria, cognitive and language abilities can also vary greatly in persons with ASD. For example, some individuals have co-occurring intellectual disabilities, while others may have average or above-average intelligence (Hulme & Snowling, 2009; Næss & Karlsen, 2022). Persons with ASD may also have additional diagnoses, such as attention deficit hyperactivity disorder (ADHD), depressive disorder, anxiety disorder and/or obsessive-compulsive disorder (BMJ Group, 2023; Helsenorge, 2023). This review employs the generic term ASD.

The global median prevalence of ASD is 65 per 10,000 (Zeidan et al., 2022). In Norway, approximately 70 per 10,000 children are diagnosed with ASD, with a notable difference between boys (110 per 10,000) and girls (30 per 10,000) (Folkehelseinstituttet, 2019). Since 2012 the prevalence of ASD has increased throughout the world, including Norway (Augustyn, 2024; Surén et al., 2019; Zeidan et al., 2022).

A significant area of interest in ASD research involves sensory processing differences, as many with ASD exhibit atypical responses to sensory stimuli—commonly referred to as hyper-responsiveness (over-sensitivity) and hyporesponsiveness (under-sensitivity) (Ashburner et al., 2008; Helsenorge, 2023; Marco et al., 2011). These responses can vary across different sensory modalities including visual input such as light and color. Importantly, the degree and type of sensory responsiveness is highly individual and can fluctuate depending on context, emotional state, or environmental conditions (Robertson & Baron-Cohen, 2017; Tavassoli et al., 2014). Furthermore, sensory processing in ASD is not static; it can be situational and circumstantial, shaped by behavioral patterns and rituals commonly observed in ASD, as well as changes in the surrounding environment. This variability makes sensory design for ASD particularly complex, as no single approach suits all individuals (Ben-Sasson et al., 2009).

Visual processing anomalies are a well-documented aspect of ASD often leading to difficulties with color perception, visual attention, and motion perception (Evers et al., 2014; Little, 2018). These can affect interaction with, and interpretation of, their surroundings, making the design of their living and learning environments even more critical. Importantly, visual stimuli often interact with other sensory modalities such as auditory or tactile modalities, intensifying or complicating individual responses. Understanding and addressing these sensory challenges is crucial especially in living and learning environments for persons with ASD (Tola et al., 2021).

Recent research underscores the significance of interior modifications in supporting persons with ASD to achieve independent living (Narenthiran et al., 2022; Wohofsky et al., 2023; Zaniboni & Toftum, 2023). A comparative study on environmental quality parameters highlighted acoustics as a critical factor, with lighting also identified as particularly significant for influencing ASD accommodations (Zaniboni & Toftum, 2023). The physical environment thus plays a crucial role in supporting sensory and behavioral needs.

We identified two literature studies on the built environment tailored for autistic persons, published in 2016 and 2022, respectively (Black et al., 2022; Martin, 2016). These two articles’ findings covered a range of topics, including lighting and color, among others. A closer examination found major limitations due to lack of the critical appraisal of the studies included in these reviews. Critical appraisal process (assessment of the risk of bias) helps to evaluate a status of the bias in design, conduct, and analysis of the study reviewed and therefore ensures quality of the data synthesized and reported (Aromataris et al., 2024). Since a critical appraisal was not performed, the translation of the findings into practical design recommendations remains limited.

The primary aim of our study was therefore to systematically review the existing literature on how indoor lighting and color affect persons with ASD and their caregivers, including a critical appraisal of the included studies. Specifically, it seeks to identify which light and color parameters can be directly utilized by designers and lighting specialists, and what objective numerical values can provide concrete guidelines to bridge the gap between explorative findings and practical application.

## Methodology

This systematic literature review investigated the impact of indoor lighting and color on persons with ASD, encompassing both children and adults, as well as their caregivers. The methodology adhered to the Preferred Reporting Items for Systematic Reviews and Meta-Analyses (PRISMA) guidelines as outlined by Page et al. (2021).

### Search

An extensive literature search was conducted across seven databases: PubMed, CINAHL, SveMed+, and four library databases including Oria, Regina, the British National Bibliography, and the Royal Danish Library. The selection of search terms for each database was guided by the PICO framework. Medical Subject Headings (MeSH) were utilized to identify appropriate keywords and relevant MeSH terms for the searches (Supplemental Appendix A). Supplemental Table 1 presents some specific English keywords employed in alignment with the PICO components.

### Eligibility Criteria

The inclusion criteria were:

1. Research involving participants diagnosed with ASD, autism, or Asperger's Syndrome, inclusive persons with concurrent visual impairments aged 0 to 100 years, as well as caregivers or educational and residential staff.
2. Studies examining the influence of lighting, color schemes, and contrast variations in interior spaces, focusing on their relationship to behavior (such as spatial navigation and daily activities) as well as user experiences and preferences.
3. Peer-reviewed journal articles published in English, Norwegian, Swedish, or Danish from 1980 onwards, corresponding to autism's formal recognition as a diagnostic category (World Health Organization, 2022).
4. Descriptive, experimental, and observational studies, using both quantitative and qualitative approaches, including self-report surveys or objective measurements.

The exclusion criteria were studies not meeting the inclusion criteria, those studies focusing exclusively on outdoor environments, and literature reviews or meta-analyses.

### Selection and Screening

**Figure 1.**
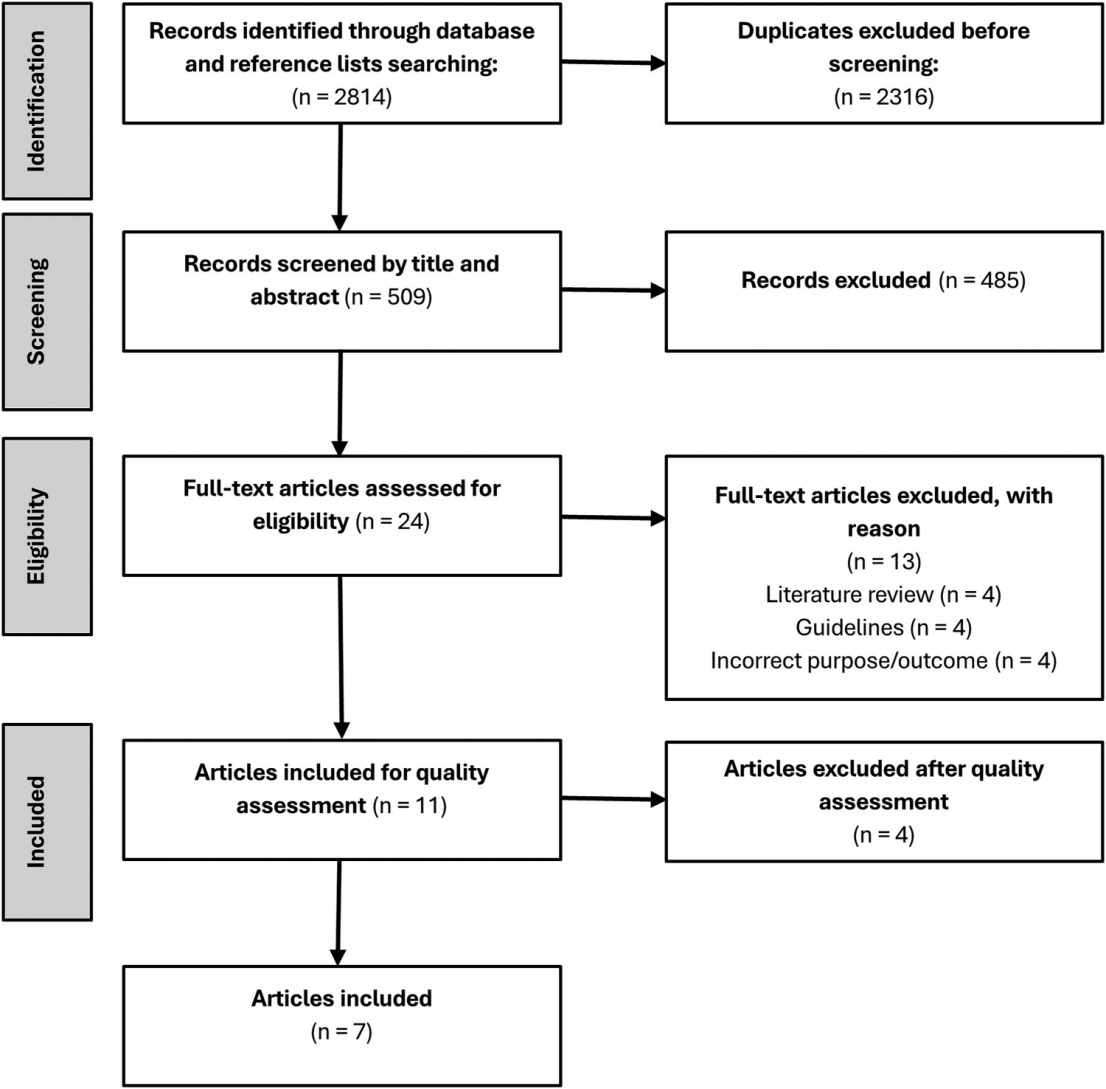
PRISMA flow chart of inclusion of articles.

Following an initial keyword database search, references were imported into EndNote, where duplicates were removed. The refined list was transferred into Rayyan QCRI (Ouzzani et al., 2016), for additional automatic and manual duplicate removal (SR). Remaining articles were screened based on titles and abstracts by three independent reviewers (SR, HKF, and HMT). Subsequently, two authors (SR and VZ) independently assessed articles against inclusion criteria, with 13% also independently reviewed by a third author (HKF). After this screening, the full-text articles were independently evaluated by two authors according to eligibility criteria. At both the title/abstract and full-text review stages, any disagreements were reconciled through discussion until consensus among all authors. The process was systematically documented using the Transparent Reporting of Systematic Reviews and Meta-Analyses (PRISMA) flow diagram, as depicted in Figure 1.

## Quality Assessment

Quality assessment of the studies was conducted using three standardized checklists, for example, JBI Critical Appraisal Checklist for Analytical Cross-Sectional Studies (Moola et al., 2017), JBI Critical Appraisal Checklist for Qualitative Research (Lockwood et al., 2015), and Mixed Methods Appraisal Tool (MMAT) (Hong et al., 2019). A guide (Glasziou & Heneghan, 2009) was used to arrive at the study design.

The traffic light approach was applied for evaluation of the risks of bias of the selected studies (Higgins et al., 2023). This approach includes five domains for assessment risk of bias (Supplemental Table 2). Questions from the selected checklists were sorted into these five domains (A1—Bias in selection of participants into the study, A2—Bias in classification of exposure, A3—Bias due to confounding, A4—Bias in measurement of outcome, and A5—Bias in the selection of the reported results; Supplemental Appendix B) and colored according to their assessed level of bias: green (low risk), yellow (moderate risk), and red (high risk). Each domain was carefully analyzed, and the overall risk of bias for each study was determined by the consensus of the authors. This method allowed for a clear and organized presentation of the bias levels across different key areas and visually represented in a table format (Supplemental Table 3) of our review.

## Results

The initial search identified 2814 articles. After removing duplicates, screening process and eligibility evaluation, this systematic review resulted in a total of 11 original articles (Figure 1 and Supplemental Table 2).

### Study Characteristics and Risk of Bias

Supplemental Table 3 shows the results of the risk of bias quality assessment (Aromataris et al., 2024). Seven out of 11 papers had low to moderate risks of bias and were included in the final analysis of this review, while four studies were excluded due to significant methodological limitations and high risk of bias. Shareef and Farivarsadri (2019) was excluded due to ambiguous aims and methodological inconsistencies (Shareef & Farivarsadri, 2019). Three other studies were also excluded due to high risk of bias (McAllister & Maguire, 2012; Nagib & Williams, 2016; Whitehurst, 2006). Detailed risk of bias quality assessment and reasons for exclusion are presented in Supplemental Table 3.

The seven remaining studies included originated from five countries, predominantly the United Kingdom (*n* = 4) and the United States (*n* = 3), with representation from Egypt (1), India (1), Australia (1), and Canada (1). These studies, published between 2006 and 2022, employed descriptive research designs: qualitative (*n* = 4), cross-sectional (*n* = 3), and mixed methods approaches (*n* = 3).

The total sample size across the studies was 2071 participants, predominantly parents, caregivers, teachers, and school staff. Participants also included autistic adults (*n* = 18), primary school students (*n* = 3), and professionals such as architects and occupational therapists (*n* = 15) with relevant experience. Questionnaires were the predominant data collection method (*n* = 7) followed by interviews (*n* = 4). Other methods included photo elicitation combined with “draw and talk” techniques (*n* = 1) and co-design involving scale-model building and computer-simulated environments (*n* = 1). Three studies specifically analyzed home or living environments, five examined classroom settings, and two did not specify the environments studied.

### Data Abstraction and Synthesis

The literature coverage analysis was conducted through thematic analysis in a deductive manner, drawing on sensory processing theory (Ashburner et al., 2008; Marco et al., 2011). Within the visual processing domain, predefined themes were outlined to explore individual responses to visual stimuli. Six themes were subsequently identified, linking lighting and color to “behavior,” “sensitivity,” and “preferences.” This thematic categorization offers a systematic overview of the varying influences that both lighting and color exert on individual responses of people with ASD across these domains.

### Thematic Analysis Results

Only the results from seven studies with either low or moderate risk of bias (high and moderate quality, Supplemental Table 3) were included in further analysis.

The identified categories/themes and the corresponding number of studies were as follows: sensitivity to lighting (*n* = 5), sensitivity to color (*n* = 4), lighting and behavior (*n* = 4), color and behavior (*n* = 3), lighting preferences (*n* = 5) and color preferences (*n* = 4), see Supplemental Table 4. While these categories are interrelated and sometimes overlap in the original studies, efforts were made to report findings in the most appropriate category based on how the phenomenon was described in the source. In presenting our results, a clear distinction was made between sensitivity, behavioral response, and preferences. Sensitivity refers to involuntary, often physiological responses to lighting or color (e.g., discomfort and overstimulation). Behavioral response refers to observable changes in activity, mood, or functioning as a reaction to the lighting or color conditions, and preference denotes subjective choices or likes.

#### Sensitivity to Lighting

Five studies identified sensitivity to lighting as an important topic and found that different lighting qualities could trigger hypersensitivity and discomfort. Williams and Vouchilas (2013) found that sensitivity to light was perceived quite frequently among their respondents with the total sensitivity percentage (45.8%) being either very sensitive or somewhat sensitive to light. Parmar et al. (2021) found discomfort associated with bright, flickering, fluorescent “strip,” and “spot” lighting. Shabha and Gaines (2013), identified intensity, light source, luminance, and windows as visual triggers, with light intensity having the strongest effect. Since no definitions for these terms, such as light intensity, were provided to the participants, the teachers’ perceptions of the terms could be different from those of design or building professionals. Gaines et al. (2014) specified fluorescent lighting, glare, brightness, flicker, and distracting windows as common sensory triggers. Among these factors, the source of lighting had the greatest impact. Nair et al. (2022) argued that flickering fluorescent lights should be avoided as they are a significant stressor, noting that subvisible flicker from these sources can cause agitation, eye strain, and headaches among children with ASD.

#### Lighting and Behavior

Four studies clearly showed that lighting affects behavior of the persons with ASD. Parmar et al. (2021) reported that alterations in lighting conditions, particularly reducing light levels, enhanced participants’ ability to function in artificially lit environments. Participants noted that intense or flickering visual stimuli could lead to fatigue or sleepiness. Nair et al. (2022) found that children often felt confused and had trouble perceiving surroundings in low light conditions. Neutral lighting was found to create a calming and soothing effect, helping to foster a more relaxing environment for them. Williams and Vouchilas (2013) described improved behavioral outcomes—such as calmer behavior, better sleep, reduced irritability, and fewer meltdowns—after lighting-related home modifications, occurring within 2 months of interventions. Gaines et al. (2014) further highlighted positive behavioral outcomes related specifically to incandescent lighting (rather than fluorescent sources), freestanding lamps instead of overhead lighting, and flexible lighting controls like dimming options.

#### Lighting Preferences

Across the reviewed studies, a clear preference emerged for natural lighting, primarily for its perceived calming properties and reduced visual triggers. Gaines et al. (2014) report that over half of respondents favored natural light for alleviating visual triggers effectively. They expressed a preference for natural light over fluorescent lighting but noted that windows could also be a distraction for students with ASD (Gaines et al., 2014). Mostafa (2008) recommends indirect natural lighting to minimize glare and distractions for hypersensitive individuals. Participants in the study by Williams and Vouchilas (2013) showed a clear preference for natural light, often using window treatments, including blackout curtains.

Artificial lighting preferences consistently pointed toward user-friendly, adjustable solutions. Dimmable and indirect lighting options (e.g., overhead lighting that is not directly visible) are preferred. Nair et al. (2022) also emphasizes the need for lights to be equipped with control switches tailored to user-specific lighting needs. LED lights are preferred over fluorescent ones due to their superior performance and less disruptive nature.

Specific preferred modifications included colored filters over fluorescent lights (Gaines et al., 2014) and replacement of fluorescent lights with incandescent or dimmable alternatives (Williams & Vouchilas, 2013). The method of “turning off all fluorescent lights” to rely on natural and incandescent light is also cited as beneficial.

There is a preference for natural and warmer color temperatures. Natural daylight color temperatures were described as comforting and calming (Parmar et al., 2021). Neutral-coloured lights are generally preferred for their less intrusive impact, as noted by Nair et al. (2022).

#### Sensitivity to Color

The reviewed studies indicate varying degrees of sensitivity to color among persons with ASD. Shabha and Gaines (2013) and Gaines et al. (2014) found that fewer than 7% of survey participants identified color and tonal contrasts of primary (floors, walls, ceilings, and doors) and secondary surfaces (furniture) as major visual triggers. Williams and Vouchilas (2013) reported minimal sensitivity among their participants, with only 6.4% indicating heightened sensitivity. Nevertheless, some studies reported more distinct sensitivity to specific colors or combinations. Findings of Zazzi and Faragher (2018) showed that environments with intense colors (e.g., combinations of blue, red, and purple) can be distracting and uncomfortable. Gaines et al. (2014) noted that bright and intense color combinations were particularly triggering. Darker shades, as reported by Nair et al. (2022), were linked to agitation and distress among autistic children. However, reactions varied significantly between individuals, highlighting the need for personalized color considerations.

#### Color and Behavior

Color adjustments demonstrated varied behavioral effects among individuals with ASD. Gaines et al. (2014) observed positive outcomes from strategically placed colors within classrooms, noting reduced undesirable behaviors when visual markers (e.g., a red poster or yellow floor tape) clearly guided actions and organized space. In contrast, Williams and Vouchilas (2013) found minimal behavioral changes associated with color-based interventions. Shabha and Gaines (2013) revealed that 15% of the United States and 6% of the U.K. teachers recognized that certain colors or contrasts could adversely affect the behavior of students with ASD. This suggests that while color can be a useful tool in some cases, its effects are not universally beneficial and can sometimes be counterproductive, depending on the individual sensitivities.

#### Color Preferences

Studies identified clear color preferences, emphasizing calming and neutral palettes for environments occupied by persons with ASD. In a qualitative study by Zazzi and Faragher (2018), interviews with three autistic children underscored a preference for calming colors in classroom settings, specifically shades of brown and white. This was similar to the findings of Gaines et al. (2014), where both focus groups and individual teachers noted a preference among students with ASD for soft, natural colors like blue and green. One educator specifically noted the benefits of gentle blue and green tones compared to neutral palettes, suggesting their ability to reduce sensory overload (Gaines et al., 2014). Nair et al. (2022) recommended pastel, muted, neutral shades as the most suitable, calming, and least distracting color choices.

Mostafa (2008) elaborated on the strategic application of colors in conjunction with lighting to cater to different sensory sensitivities. The study described the use of bright colors to stimulate individuals with hyposensitivity and neutral colors to provide a calming atmosphere for those with hypersensitivity. Additionally, warm colors were recommended to convey psychological warmth to individuals experiencing hypotactile sensitivity, indicating a nuanced understanding of how different color tones can meet diverse sensory needs.

#### Other important findings

An important new finding identified by our study is the connection between indoor daylight sensitivity and seasonal/climatic variations (Parmar et al., 2021; Shabha & Gaines 2013). Shabha and Gaines (2013) suggested that differences in participant responses between the U.S. and U.K. groups might be attributed to regional variations in climate and the amount of daylight availability indoors. Importantly, Parmar et al. (2021) highlighted sleeping difficulties experienced during summer months was likely due to prolonged daylight hours. Another interesting finding was that the interaction of light and color could have positive effects (such as improved comfort and calmness) on autistic children (Nair et al., 2022).

## Discussion and Conclusions

The aim of this study was to systematically review published research on how indoor lighting and color affect persons with ASD, specifically to critically assess the quality, identify relevant lighting and color parameters, and, if possible, to extract measurable values useful for practical application by designers.

This review provided significant insights into the sensitivity of autistic individuals to various lighting conditions. Four of the seven studies meeting quality criteria identified specific lighting attributes, such as intensity, luminance, glare, daylight, flicker, and especially type of light source (fluorescent, incandescent, and LED), as triggers for hypersensitivity. However, a critical limitation across these studies was the lack of precise, objective measurement of lighting parameters. Consequently, it is impossible to determine the exact levels of light intensity or glare that triggered negative reactions. While standard classroom light levels might be inferred, reconstructing the light conditions in residential settings is impractical. The only clearly identified and consistently problematic parameter was fluorescent lighting.

Despite consensus on lighting's behavioral impact, contradictions emerged across observational findings. Parmar et al. (2021) reported that lower light levels improved visual capacity for ASD persons, whereas Nair et al. (2022) found that low light levels hindered the ability to observe surroundings. These discrepancies are difficult to compare objectively due to the lack of light measurements and the subjective nature of light perception.

Several positive behavioral improvements were also documented, such as the calming effects of neutral lighting (Nair et al., 2022), the preference for incandescent lamps over fluorescent lights, and the use of freestanding lamps with dimmers (Gaines et al., 2014). While these lighting improvements are practical to implement, recommendations regarding light levels and “neutral lighting” remain vague without specific, applicable guidelines.

Key findings across the reviewed studies emphasize the importance of natural daylight as the primary source of illumination in spaces for autistic persons (Gaines et al., 2014; Mostafa, 2008; Williams & Vouchilas, 2013). These preferences highlight the need for adaptable, personalized lighting designs to enhance both comfort and functionality, particularly in educational and therapeutic contexts. Additionally, strategic color choices in classroom settings were shown to potentially improve behavioral and educational outcomes, although these must be tailored due to the varying sensitivity among individuals with ASD.

Our results align thematically with several findings from the literature reviews of Martin (2016) and Black et al. (2022). Both studies highlighted the benefits of minimizing color variation in classroom interiors to support calmness, the use of covered windows or clerestory windows to minimize distractions, the combination of natural and electrical lighting, the necessity of controlling glare and overheating from direct sunlight, and the importance of individual lighting control and a range of task lighting options. However, a closer examination of Martin's (2016) review revealed notable methodological limitations. Of the 20 sources included, seven were secondary research papers. Among the remaining 13, only nine focused specifically on autism or ASD, and just three addressed lighting quality—with only one of these being a peer-reviewed study directly examining lighting and ASD (McAllister, 2010; McAllister & Maguire, 2012). This limited the reliability of the conclusions drawn in that review. Black et al. (2022) reinforced previous findings, advocating for reduced light intensity to avoid hyperstimulation, the preference for dimmable and easily controllable lighting, and the use of daylight while restricting distracting views. They also recommended using clerestory windows, avoiding glare and direct sunlight, adjustable window shades, indirect lighting, and cove lighting. Despite these recommendations, the study also highlighted controversial conclusions, such as the avoidance of overhead lighting and the beneficial use of skylights. Although both reviews provide useful thematic insights, the absence of consistent, rigorous quality assessment limits the strength of their conclusions. While our current findings may thematically align with those reported earlier, this convergence likely reflects the recurring nature of certain design considerations across the field rather than a validation of methodological robustness.

Our analysis revealed a gradual increase in published original research; however, most studies remain exploratory and observational. The consistent findings across previous and current studies demonstrated the reliability of the evidence but also revealed a lack of significant progress in the field. The persistent lack of practical, numeric guidelines significantly restricts designers’ ability to translate research into practice. Given that sensory experiences, behavioral reactions, and preferences for light and color vary significantly among individuals with ASD, future research should adopt more rigorous designs that group participants based on their sensory responsiveness such as hyper- or hyposensitivity. Rather than attempting to create universal or “one-size-fits-all” recommendations, research should focus on identifying distinct subgroups within the neurodiverse population. By systematically categorizing participants according to their sensory profiles and investigating their specific environmental responses, researchers can develop targeted, evidence-based lighting and color guidelines tailored more precisely to different sensory needs. Such an approach would also help to identify which design solutions might be suitable or unsuitable for both subgroups, highlighting strategies beneficial across sensory profiles or those consistently problematic for them. This would offer clearer and more practical guidance for designers and practitioners compared to generalized recommendations alone.

Our results reflect a general neglect of lighting design for the neurodiverse population within the architectural and lighting design communities. Although the number of studies is slowly increasing, the absence of lighting experts in research teams hampers the development of sufficient evidence for proper lighting design. This contributes to the exclusion of neurodiverse individuals from public life and their right to access supportive and inclusive environments, including educational settings.

An important yet often overlooked aspect in inclusive design is the interaction between lighting and color. Lighting significantly influences how colors are perceived (Boyce, 2014), but this relationship was not systematically addressed in the reviewed studies, suggesting it is undervalued or overlooked by researchers. Since color recommendations cannot be effectively applied without considering the lighting context, designers should integrate lighting and color choices carefully to support sensory comfort. Future research should explicitly address this critical gap.

Methodological limitations of the reviewed studies were evident. Many studies relied predominantly on qualitative surveys or interviews—without systematically documenting environmental variables (e.g., illuminance levels, correlated color temperature, and glare). Additionally, direct involvement of individuals with ASD in the research process was limited. Future studies must employ validated, rigorous methodologies, encompassing detailed environmental data collection and direct user involvement, to generate evidence-based, tailored environmental modifications for diverse sensory profiles.

In conclusion, while the current body of research provides valuable insights (e.g., daylight preferences, flexible lighting controls, reduced glare, warmer colors, and tailored lighting solutions) significant gaps remain. Addressing these gaps through rigorous, precise, and methodologically robust research is essential to produce practically applicable guidelines that foster supportive environments for the neurodiverse population.

## Implications for Practice

The research findings outlined in the article provide valuable insights into the effects of lighting and color on individuals with Autism Spectrum Disorder (ASD), suggesting several practical applications:

- **Enhancing environments for sensory sensitivity** through proper design solutions that help to minimize hypersensitivity triggers by for example avoiding bright, flickering, and fluorescent lights, which are known to cause discomfort and agitation; control of the light intensity; use of natural lighting to reduce sensory overload and others.
- **Understanding** that persons with ASD may require tailored lighting for their comfort and strategic use of color to mitigate behavioral issues.
- **Educating designers and stakeholders** in understanding the profound impact that lighting and color have on individuals with ASD, ensuring that environments are designed from the outset to accommodate these needs, potentially reducing the need for later modifications.

## Supplemental Material

sj-docx-1-her-10.1177_19375867251373096 - Supplemental material for Systematic Literature Review: Indoor Lighting and Color Effects on Persons With ASD Supplemental material, sj-docx-1-her-10.1177_19375867251373096 for Systematic Literature Review: Indoor Lighting and Color Effects on Persons With ASD by Veronika Zaikina, PhD, Hanne-Mari Schiøtz Thorud, PhD, Susanne Færdi Rustad, MSc, and Helle Kristine Falkenberg, PhD in HERD: Health Environments Research & Design Journal

sj-docx-2-her-10.1177_19375867251373096 - Supplemental material for Systematic Literature Review: Indoor Lighting and Color Effects on Persons With ASD Supplemental material, sj-docx-2-her-10.1177_19375867251373096 for Systematic Literature Review: Indoor Lighting and Color Effects on Persons With ASD by Veronika Zaikina, PhD, Hanne-Mari Schiøtz Thorud, PhD, Susanne Færdi Rustad, MSc, and Helle Kristine Falkenberg, PhD in HERD: Health Environments Research & Design Journal
