# Supplementary material for: Systematic Literature Review: Indoor Lighting and Color Effects on Persons With ASD
Source: HERD. 2025 Oct 23;19(1):224–36. doi: 10.1177/19375867251373096 (PMC12715023; doi:10.1177/19375867251373096)
Supplement: sj-docx-1-her-10.1177_19375867251373096 - Supplemental material for Systematic Literature Review: Indoor Lighting and Color Effects on Persons With ASD [file sj-docx-1-her-10.1177_19375867251373096.docx]

## **Appendix A**

MeSH terms utilized for conducting searches in different languages.

| Intervention | | Population | | Comp. | Outcome | |
| --- | --- | --- | --- | --- | --- | --- |
| English | Norwegian/ Danish | English | Norwegian/ Danish |  | English | Norwegian/ Danish |
| Disorder, Autistic  Disorders, Autistic  Disabilities, Intellectual  Intellectual Disabilities  Development Disorder, Intellectual  Development Disorders, Intellectual  Disorder, Intellectual Development  Disorders, Intellectual Development  Disability, Intellectual  Vision Disorder  Visual Disorders  Disorder, Visual  Disorders, Visual  Visual Disorder  Visual Impairments  Vision Disability  Disabilities, Vision  Disability, Vision  Vision Disabilities | Autistisk forstyrrelse  Barneautsme  Infantil autisme  Lysoverfølsomhet  Synsskader  Synsnemning  Nedsatt syn  Redusert synsevne  Svakt syn  Svekket syn  Synsnedsettelse  Synssvekkelse | Light, Visible  Visible Light  Daylight Vision  Vision, Daylignt  Colors  Color perceptions  Perception, Color  Perceptions, Color  Color Visons  Vision, Color  Visions, Color  Interior Furnishings  Furnishing, Interior  Furnishings, Interior  Furnishing, Interior  Interior Furnishing  Interior Design  Design, Interior  Designs, Interior  Interior Designs | Synlig lys  Lys, synlig  Dagslyssyn  Fargeoppfatning  Fargeoppfattelse |  | Navigation, Spatial  Navigations, Spatial  Spatial Navigatons  Spatial Visualization  Spatial Visualizations  Visualization, Spatial  Visualizations, Spatial  Spatial Ability  Abilities, Spatial  Ability, Spatial  Spatial Abilities  Orientations, Spatial  Spatial orientation  Spatial Orientations  Behaviour, Spatial  Behaviours, Spatial  Spatial Behaviours  Processing, Spatial  Processings, Spatial  Spatial Processings  Visual-Auditory Spatial Processing  Processing, Visual- Auditory Spatial  Processings, Visual- Auditory Spatial  Spatial processing, Visual-Auditory  Spatial processings, Visual-Auditory  Visual Auditory Spatial Processing  Visual-Auditory Spatial Processings  Auditory-Visual Spatial Processing  Auditory Visual Spatial Processing  Auditory-Visual Spatial Processings  Processing Auditory-Visual Spatial  Processings, Auditory-Visual Spatial  Spatial Processing, Auditory-Visual  Spatial Processings, Auditory-Visual  Visual Spatial Processing  Processing, Visual Spatial  Processings, Visual Spatial  Spatial Processing, Visual  Spatial Processings, Visual  Visual Spatial Processings  Memories, Spatial  Memory Spatial  Spatial Memories | Romlig navigasjon  Spatial orientering  Romlig atferd  Romlig prosessering  Romlig minne |
