# Supplementary material for: Systematic Literature Review: Indoor Lighting and Color Effects on Persons With ASD
Source: HERD. 2025 Oct 23;19(1):224–36. doi: 10.1177/19375867251373096 (PMC12715023; doi:10.1177/19375867251373096)
Supplement: sj-docx-2-her-10.1177_19375867251373096 - Supplemental material for Systematic Literature Review: Indoor Lighting and Color Effects on Persons With ASD [file sj-docx-2-her-10.1177_19375867251373096.docx]

## **Appendix B**

| **Tool / Q&A Risk of Bias Areas** | **Questions** | | | | | **Answers** | |
| --- | --- | --- | --- | --- | --- | --- | --- |
|  | **A1 - Bias in selection of participants into the study** | **A2 - Bias in classification of exposure** | **A3 - Bias due to confounding** | **A4 - Bias in measurement of the outcome** | **A5 - Bias in the selection of the reported results** | **A1-A5** | **Overall risk of bias** |
| **JBI (cross-sectional)** | 1. Inclusion criteria | 3. Exposure measurement | 5. Confounding factors | 7. Outcome measurement | 8. Statistical analysis | Yes/No/ Unclear | Low: ‘yes’ on almost all questions  Moderate: mix of ‘yes’, ‘no’, and ‘unclear’  High: ‘no’, or ‘unclear’ on many/all questions |
|  | 2. Study subjects and settings |  | 6. Strategies for confounding factors |  |  |  |  |
|  | 4. Measurement criteria |  |  |  |  |  |  |
| **JBI (qualitative)** | 6. Theoretical/ cultural background | 1. Theoretical perspective and method | Not applicable | 4. Data analysis and method | 6. Theoretical/ cultural background | Yes/No/ Unclear | Low: ‘yes’ on almost all questions  Moderate: mix of ‘yes’, ‘no’, and ‘unclear’  High: ‘no’, or ‘unclear’ on many/all questions |
|  | 7. Researcher’s influence | 2. Research question and method |  | 5. Results interpretation and method | 7. Researcher’s influence |  |  |
|  | 9. Ethical approval | 3. Data collection and method |  | 6. Theoretical/ cultural background | 8. Representation of participants |  |  |
|  |  | 6. Theoretical/ cultural background |  | 7. Researcher’s influence | 10. Conclusion of reported findings |  |  |
|  |  | 7. Researcher’s influence |  |  |  |  |  |
| **MMAT** | 4.1 Quantitative descriptive: sampling strategy and research question | S1. Research question | 5.5 Components and different methods | 1.5 Qualitative: sources, collection, analysis, interpretation | 1.3 Qualitative: data extraction | Yes/No/  Can’t tell | Low: ‘yes’ on almost all questions  Moderate: mix of ‘yes’, ‘no’, and ‘unclear’  High: ‘no’, or ‘unclear’ on many/all questions |
|  |  | S2. Data collection and research question |  | 4.3 Quantitative descriptive: measurements | 1.4 Qualitative: results interpretation |  |  |
|  | 4.2 Quantitative descriptive: sampling representation | 1.1 Qualitative: method and research question |  | 4.4 Quantitative descriptive: nonresponse bias | 5.3 Interpretation |  |  |
|  |  | 1.2 Qualitative: data collection method and research question |  | 4.3 Quantitative descriptive: statistical analysis and research question | 5.4 Inconsistent results |  |  |
|  |  | 5.1 Mixed methods and research question |  |  |  |  |  |
|  |  | 5.2 Components and research question |  |  |  |  |  |
